# Verbal Memory Impairment in Polydrug Ecstasy Users: A Clinical Perspective

**DOI:** 10.1371/journal.pone.0149438

**Published:** 2016-02-23

**Authors:** Kim P. C. Kuypers, Eef L. Theunissen, Janelle H. P. van Wel, Elizabeth B. de Sousa Fernandes Perna, Anke Linssen, Anke Sambeth, Benjamin G. Schultz, Johannes G. Ramaekers

**Affiliations:** Department of Neuropsychology & Psychopharmacology, Faculty of Psychology & Neuroscience, Maastricht University, Maastricht, The Netherlands; University of Granada, SPAIN

## Abstract

**Background:**

Ecstasy use has been associated with short-term and long-term memory deficits on a standard Word Learning Task (WLT). The clinical relevance of this has been debated and is currently unknown. The present study aimed at evaluating the clinical relevance of verbal memory impairment in Ecstasy users. To that end, clinical memory impairment was defined as decrement in memory performance that exceeded the cut-off value of 1.5 times the standard deviation of the average score in the healthy control sample. The primary question was whether being an Ecstasy user (E-user) was predictive of having clinically deficient memory performance compared to a healthy control group.

**Methods:**

WLT data were pooled from four experimental MDMA studies that compared memory performance during placebo and MDMA intoxication. Control data were taken from healthy volunteers with no drug use history who completed the WLT as part of a placebo-controlled clinical trial. This resulted in a sample size of 65 E-users and 65 age- and gender-matched healthy drug-naïve controls. All participants were recruited by similar means and were tested at the same testing facilities using identical standard operating procedures. Data were analyzed using linear mixed-effects models, Bayes factor, and logistic regressions.

**Results:**

Findings were that verbal memory performance of placebo-treated E-users did not differ from that of controls, and there was substantial evidence in favor of the null hypothesis. History of use was not predictive of memory impairment. During MDMA intoxication of E-users, verbal memory was impaired.

**Conclusion:**

The combination of the acute and long-term findings demonstrates that, while clinically relevant memory impairment is present during intoxication, it is absent during abstinence. This suggests that use of Ecstasy/MDMA does not lead to clinically deficient memory performance in the long term. Additionally, it has to be investigated whether the current findings apply to more complex cognitive measures in diverse ‘user categories’ using a combination of genetics, imaging techniques and neuropsychological assessments.

## Introduction

The party drug Ecstasy was highly popular in the 1980’s and 1990’s and its use peaked in mid-2000, after which it declined. Research in the Netherlands has shown that the percentage of ecstasy tablets containing an MDMA-like substance was relatively high and stable (80 to >95%) between 1999–2008 whereas it declined rapidly from 2008 on, a phenomenon also seen in other EU countries [1]. Recent data has shown that the scenery has changed again with a higher availability of pills with a high dose of MDMA and MDMA in powder- or crystal form [2]. The Drug Information and Monitoring System in the Netherlands showed that while ecstasy tablets up to 2009 contained less than 100 mg MDMA per tablet, this increased over the last years with more higher dosed tablets (100 to > 150 mg MDMA) [3, 4].

As of 2015, Ecstasy use stabilized but annually still accounts for 1–3% of the general population reported use in Europe, the US, and Australia demonstrating that a substantial proportion of the population comes in contact with this substance once in their lives [2, 5–7]. It is known that the percentage of E-users is higher in particular social settings. It has been shown in a study amongst regular night clubbers that 37% of them used Ecstasy in the past year [2]. Smirnov et al. showed that young people’s Ecstasy use in general is relatively transient, that is, a majority of young adult E-users consume Ecstasy relatively infrequently and have declining levels of use before reaching their mid-twenties [8].

Ever since Ecstasy became popular, researchers have tried to uncover potential long lasting negative effects of the drug on mental health and cognition (e.g., [9]). Of particular concern has been the memory impairment, observed in abstinent (heavy) Ecstasy or E-users (e.g., [10–14]). In general, two sources of evidence show that there is a relation between Ecstasy/MDMA use and memory impairment. Firstly, placebo-controlled studies have shown that a single dose of MDMA (75 mg) reduces verbal memory performance 1.5 hours after administration, as indicated by a lower recall performance on an immediate and delayed recall test [15–19]. Controlled MDMA administration studies also demonstrated that acute memory impairment was transient in nature, that is, verbal memory performance was equal to placebo performance, 25.5 hours after MDMA administration [18]. Together these results indicate that MDMA is able to temporarily distort verbal memory in light E-users and that a single dose in those users does not lead to irreversible verbal memory impairment.

Secondly, retrospective studies in Ecstasy users have shown that these acute effects might become chronic after long term use. Accordingly, Ecstasy use would cause impairments in memory performance and/or changes in brain activation and structures that are important for memory encoding and retrieval compared to control subjects (e.g. Ecstasy-naïve polydrug users, cannabis users, or drug-naïve participants) ([20–23]; c.f. [24–26]). A meta-analysis by Nulsen et al. (2010) showed that E-users performed significantly worse on short-term verbal memory tasks compared to drug-naïve and polydrug controls. Short-term memory performance was not predicted by life-time Ecstasy consumption which was between 1 and 1000 tablets in total [14].

Generally, these retrospective study designs encounter a number of methodological issues such as polydrug use, impurity of tablets and unknown dose, reliability of self-reported drug history, and (premorbid) group differences that may impede comparisons between groups and interpretations of results [26, 27]. A number of these issues can be circumvented by prospective studies in which a group of drug-naïve persons is followed in time and tested several times before and after onset of Ecstasy use. Thus, it could be determined whether differences in cognitive performance between E-users and non-users emerged before or after the onset of MDMA use. In the ‘NeXT’ (the Netherlands XTC Toxicity) study, persons with a high probability to start using Ecstasy, as indicated by the intention to probably or certainly use Ecstasy in the near future, were included [28, 29]. Schilt and colleagues (2007) showed that difference scores of verbal memory between baseline and follow-up measures (within three years) were significantly lower in the novice E-users (average lifetime use: 3.2 tablets) compared to the persistent Ecstasy-naïve persons [29]. However, Krebs rightly remarked that the findings of this nonrandomized study were overstated as all test scores were within the normal range. Moreover, the E-users did not deteriorate over sessions but the control group improved. Attention was also drawn to the fact that some of the ‘novice’ users had used ten times more than the group average and it could, therefore, not be qualified as low/light use. Furthermore, there were no available data on the dose itself, and high doses cannot be excluded [30]. In another prospective study, no effects of Ecstasy use on verbal memory were shown. In this study, however, the inclusion criterion ‘a high probability of future ecstasy use’ was, in contrast to Schilt et al., operationalized as having first-hand but very limited experience with MDMA. The group was split in two, based on the continuation of Ecstasy/MDMA use in the year following the baseline assessment [31].

Despite these methodological differences between different prospective studies, and between prospective versus retrospective studies, the results seem to point in the same direction. A systematic review of observational evidence concluded that, while verbal memory performance of E-users differs from that of control subjects, the scores tend to fall within normal ranges, that is, there is no solid evidence of clinical significance, and deficits are likely to be small [24]. While a meta-analysis of this kind provides valuable evidence, it encounters some difficulties like heterogeneity in study design and measures, and unequal distribution of potential confounders in the study arms (e.g., age) [24]. In order to overcome these issues, the present data analysis used linear mixed-effects models (LMEM) to account for variability that might be introduced by confounds, such as, age, sex, and study (including unknown variations in experimenters, recruitment style, and population from which the sample was recruited).

The strength of the present study is that, in contrast to a common meta-analysis, this study pooled the individual data from studies that used identical standard operating procedures at identical test locations, same study design (e.g., placebo-controlled), using the same assessment of memory performance (i.e., the Word Learning Task)[15–17, 19, 32–35]. In addition, combining these datasets allowed the assessment of both the acute and long-term effects of MDMA use in a large sample of E-users.

The main aim was to investigate the ‘long-term’ or chronic effects of low Ecstasy use on memory performance. The research question was whether light polydrug E-users demonstrated a great frequency of clinically relevant memory impairment than a healthy control group. To that end, a ‘verbal memory impairment’ criterion was calculated that was used to signal clinical significance of the scores. Based on the literature about ‘Mild Cognitive Impairment’ (MCI), objective memory impairment is defined as performance below a cut off of 1.5 standard deviations of the average score in a healthy control sample [36, 37]. Although this criterion is frequently used in clinical neuropsychology to indicate clinical neuropsychological deficits, it doesn’t imply that this cutoff is determinative with respect to functional abilities in daily life. Interestingly, studies of healthy elderly samples have shown that performance in immediate verbal memory was the best predictor for activities of daily living [38]. Furthermore, it was also demonstrated that patients with MCI, performing minimally 1.5 SD below the norm, do have functional impairments compared to a healthy control group [39].

Based on previous studies on E-users, it is hypothesized that, compared to a healthy control group, E-users show lower memory scores. However, it was expected that the clinical significance of these findings (e.g. functional impairment in daily life) was low as also suggested by another meta-analysis [24]. Additionally, it was investigated whether quantifiers of Ecstasy/MDMA exposure (i.e., ‘times used’ and ‘number of years of use’) contribute significantly to long-term effects on memory performance as defined by clinically relevant memory deficits. ‘Short-term’ or acute effects of MDMA administration on clinical relevance of memory impairment were also studied. Furthermore, it was investigated whether MDMA disposition (MDMA blood concentrations) predict clinically deficient memory performance.

## Materials and Methods

### Participants

Two groups were included in the present study: Polydrug E-users from four placebo-controlled experimental MDMA studies [15–17, 19] conducted between 2008 and 2012, and drug-naïve participants from six placebo-controlled experimental studies investigating the acute effects of medicinal drugs on cognition [33–35]. These ten separate studies were all approved by the Medical Ethics Committee of Maastricht University and the University Hospital.

The pooled sample size from the MDMA studies was 70; five participants, however, participated in multiple MDMA studies so for them, only the first participation was included in the analysis. The remaining 65 participants were matched by age and gender. In case there were more matching options, the match was chosen randomly. In case there was no matching option, the participant with the nearest age was randomly chosen so that the average age of both samples was equal. Demographic details of E-users and matched controls are given in Table 1. Drug use history (Table 2) was checked with a medical questionnaire and through an interview with a medical doctor. The control participants were drug naïve and did not excessively use alcohol (less than 20 alcoholic consumptions per week) as per the inclusion criteria. Inclusion and exclusion criteria of all studies were identical apart from the drug use whereby a history of illicit drug use resulted in the exclusion of participants from the control group.

**Table 1:** Demographic details of Ecstasy users and matched controls.

	E-users	Matched Controls
Age		
Mean (SD)	21.68 (2.11)	21.71 (2.13)
Min	18	18
Max	28	28
Gender		
M:F	40:25	40:25
Education level		
Missing data (%)	27.7	9.2
% related to non-missing data		
- University students	38.3	47.8
- Non-University students	61.7	52.2

**Table 2:** Drug use history (number of times used in lifetime) E-users.

Substance	Min	Max	Mean (SD)	Used (N)	Never used (N)	Missing data (N)
Ecstasy/MDMA (number of times in lifetime)	1	780	30.28 (99.83)	64	n.a.	1^¶^
Ecstasy/MDMA (duration of use in years)	1	12	3.69 (2.24)	62	n.a.	3
Alcohol	60	2600	590.74 (592.58)	63*	-	2**
Amphetamine	1	49	10.71 (14.20)	15†	50	n.a.
Cannabis	1	3650	236.18 (675.68)	57°	8	n.a.
Cocaine	1	40	13.61 (11.81)	24^§^	41	n.a.
LSD	1	2	1.33 (0.58)	3	62	n.a.
Mushrooms/ ‘Magic Truffles’ (psychoactive substance: psilocybine, psilocine)	1	50	4.15 (8.32)	36^$^	29	n.a.
Other:						
- 2CB	2	2	2.00	1	64	n.a
- GHB (1: 20 times, other: no info)	-	-	-	2	63	n.a
- Ketamine	5	10	7.50 (3.53)	2	63	n.a
- Salvia	1	1	1.00	1	64	n.a

^¶^ One participant stated using Ecstasy/MDMA ‘sometimes’ without putting it in numbers;

*36 participants did not quantify their use in numbers but stated to use alcohol on a ‘weekly’ base (N = 7), claimed that they use it ‘often’ (N = 17), defined their use as ‘average’ (N = 3), or stated using alcohol without putting it in numbers or quantifying it in words (N = 9);

**data about alcohol use was missing for 2 participants;

^†^ 1 participant stated using amphetamines without putting it in numbers or quantifying it in words;

° 13 participants did not quantify their use in numbers but stated to use cannabis ‘once in a while’ (N = 2), ‘often’ (N = 5), ‘rarely’ (N = 1), ‘+100 times’ (N = 1), or stated using it without putting it in numbers or quantifying it in words (N = 4);

^§^ One participant stated using cocaine without putting it in numbers or quantifying it in words;

^$^ Two participants stated using ‘Magic Truffles’ without putting it in numbers or quantifying it in words

Participant recruitment in all studies was conducted by means of advertisements in the buildings of Maastricht University, in local newspapers, an internet site (digiprik), and by word of mouth.

### Procedure

After enrolment in the separate studies, participants did a training session during which they were familiarized with tests and test procedures. On the day prior to each test day, the use of alcohol was prohibited. E-users were also asked to refrain from any drug use at least one week prior to the medical screening until the last test day. Participants were instructed to arrive well-rested (following a normal night’s sleep) at the test facilities; this was assessed by means of a self-rated sleep scale. Participants were screened for alcohol and drug use in breath and urine respectively at the start of a test day. When tests were negative they were allowed to proceed with the test day. After a light breakfast and a mood questionnaire, participants in all studies were given their ‘treatments’ (either active substance or placebo). At peak drug concentrations the cognitive tests started. In all studies, the first neuropsychological assessment was the Word Learning Task Immediate Recall (WLT-IR) test followed by 30 minutes of tasks after which the Word Learning Task Delayed Recall (WLT-DR) test was conducted. In all the included experimental MDMA studies, blood was drawn 1.5 hours after MDMA (75 mg) and placebo administration, that is, prior to the WLT. For a detailed description of the toxicological determination, see [15–17, 19].

### Word Learning Task

All of the studies included in this analysis used the same adapted version and procedure of the Word Learning Task (WLT) [40]. There were parallel versions for separate test days. The WLT consisted of thirty Dutch mono-syllabic meaningful nouns and adjectives that were consecutively presented on a computer screen [16, 40]. Participants were to verbally recall as many words as possible (immediate recall). This procedure was repeated three times; immediate scores were summed to comprise the Total Immediate Recall score. After a 30-minute delay participants were asked to recall as many of the previously learnt words as possible (delayed recall). The dependent variables used for these analyses were the Total Immediate Recall (IR) Score and the Delayed Recall (DR) Score.

### Verbal Memory Impairment Criterion

A ‘verbal memory impairment’ criterion was calculated from age- and gender-matched verbal memory performance data of the control sample. This cut off of objective impairment, based on the literature on ‘Mild Cognitive Impairment’ was less than 1.5 standard deviations of the average score in a healthy control sample [36, 37]. Using this criterion based on the scores of the control group, scores in both the control group and the E-users group were classified into two categories: impaired or not.

### Statistical analysis

A Linear Mixed-Effects Model (LMEM) was performed to examine differences in verbal memory ability (i.e., immediate and delayed recall scores) between MDMA users and controls. The LMEM approach was used to account for variability that may have been introduced by age, sex, and study. The LMEM was fit to the data with fixed factor group (two levels: MDMA user, control) and random factors study (ten levels), sex (two levels), and age (continuous) where sex and age were nested within study (i.e., using the maximal random effects structure justified by the experimental design, following [41]). A second LMEM was used to compare E-users on the within-subjects fixed factor MDMA Treatment (two levels: MDMA, Placebo) and the same random effects. The model was fit using the *lme* function of the *nlme* library [42, 43] for the R package of statistical computing [44]. A separate LMEM was used to analyze each of our dependent variables (immediate recall and delayed recall). F-statistics, significance values, and effect sizes (generalized eta^2^) were calculated using Satterthwaite approximation for degrees of freedom.

As classical null-hypothesis testing statistics are not designed to find evidence for an absence of difference between conditions, the JZS Bayes factor was calculated to test the hypothesis that MDMA users and controls do not differ in their immediate or delayed recall ability. The JZS Bayes factor quantifies the strength of evidence in favour of the null hypothesis (when *BF*_*01*_ < 1) or in favor of the alternative hypothesis (when *BF*_*01*_ > 1; [45]). The Bayes factor was computed using the *ttestBF* function for paired-samples designs in the *BayesFactor* library [46]. LMEM and Bayes factor analyses were performed using R statistical software (version 3.2.2).

Regression analyses were performed to determine relationships between drug use and verbal memory performance. Several binary logistic regressions were conducted in order to test the contribution of several factors to the chance of having clinical memory deficits immediately after MDMA use, or in the long-term. The dependent variable was ‘verbal memory impairment’ (yes/no); single predictors in the 5 separate analyses were ‘Ecstasy user’ (yes/no), times used Ecstasy (number), years used Ecstasy (number), MDMA intoxication (MDMA/placebo), MDMA blood concentration (μg/L). Odds ratio (OR) together with 95% Confidence Intervals (CI) are reported. Regression analyses were conducted using the statistical software package SPSS (version 21.0). The significance level (alpha) for all tests was set at 0.05.

## Results

### Missing data

With regard to the behavioural data we had 65 complete sets for the IR-Score, and only 48 complete sets for the DR-Score since these data were not collected in one of the MDMA studies [19]. Furthermore, data about life time Ecstasy use (times used/years of use) were missing for four participants (one for ‘times used’; three for ‘years of use’), and MDMA concentrations were missing for six participants. In case of missing data, specific analyses were run only including complete data sets. WLT data of the 10 separate studies are shown in Table 3.

**Table 3:** Mean and standard deviation of immediate recall (IR) and delayed recall (DR) in the word learning task for each study.

Study	E-user Control	*N*	Immediate RecallMean (SD)	Delayed RecallMean (SD)	N with IR impairment	N with DR impairment
1	E	17	38.88 (11.42)	-	1	0
2	E	17	42.47 (10.67)	13.94 (5.54)	1	0
3	E	16	38.87 (8.80)	12.25 (5.67)	2	1
4	E	15	54.07 (14.75)	20.07 (6.58)	1	0
5	C	6	37.00 (9.46)	10.00 (3.79)	0	0
6	C	7	40.14 (15.54)	13.86 (7.51)	0	0
7	C	8	54.00 (8.63)	21.12 (4.12)	0	0
8	C	13	41.69 (9.23)	13.85 (5.10)	0	0
9	C	14	52.93 (12.41)	18.79 (6.88)	2	1
10	C	17	44.82 (12.72)	15.82 (5.09)	0	1

### Long-term or chronic effects of Ecstasy use

#### E-users versus healthy controls

**Fig 1:**
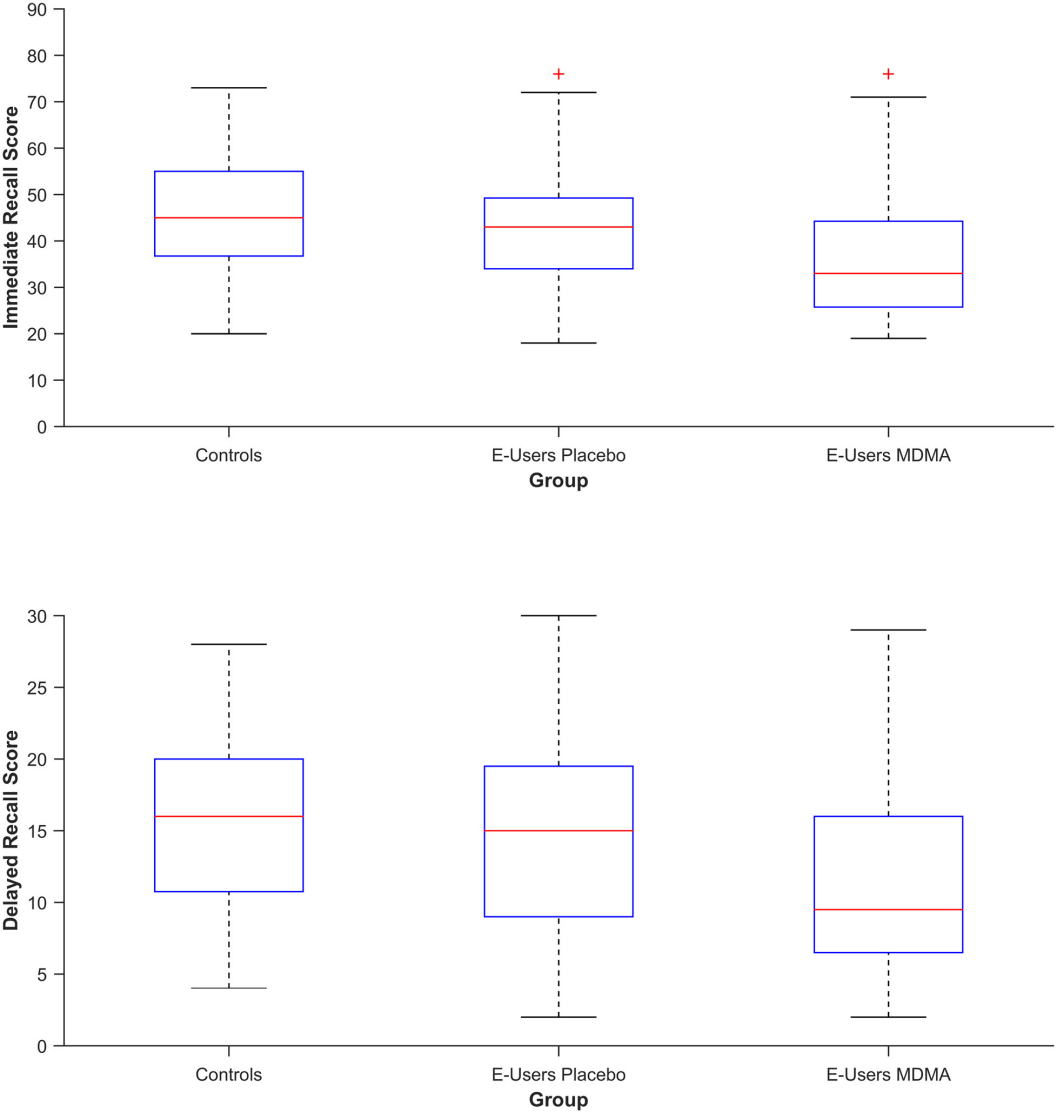
The median (red line), 25th and 75th percentiles (box edges), range excluding outliers (whiskers), and outliers (red +) of the Total Immediate Recall (IR) Score (upper graph) and Delayed Recall (DR) Score (lower graph) of Controls, and Ecstasy users (E-Users) in the placebo condition and MDMA condition.

The LMEM yielded no significant differences between E-users and healthy controls on measures of immediate recall [*F* (1, 9.08) = 0.24, *p* = .64, *η*^*2*^*G* = .01] and delayed recall [*F* (1, 7.65) = 0.02, *p* = .89, *η*^*2*^*G* = .0004] (Fig 1, placebo scores). To test whether there was evidence in favor of the null hypothesis for differences between E-users and controls, the Bayes factor was calculated. There was substantial evidence for the null hypothesis (see Jeffreys, 1961) for the immediate recall (*BF*_*01*_ = 4.36 ± 0%) and delayed recall (*BF*_*01*_ = 4.55 ± 0%) tasks. These results indicate that long-term MDMA use does not impair verbal memory ability.

#### Ecstasy use and memory performance

**Fig 2:**
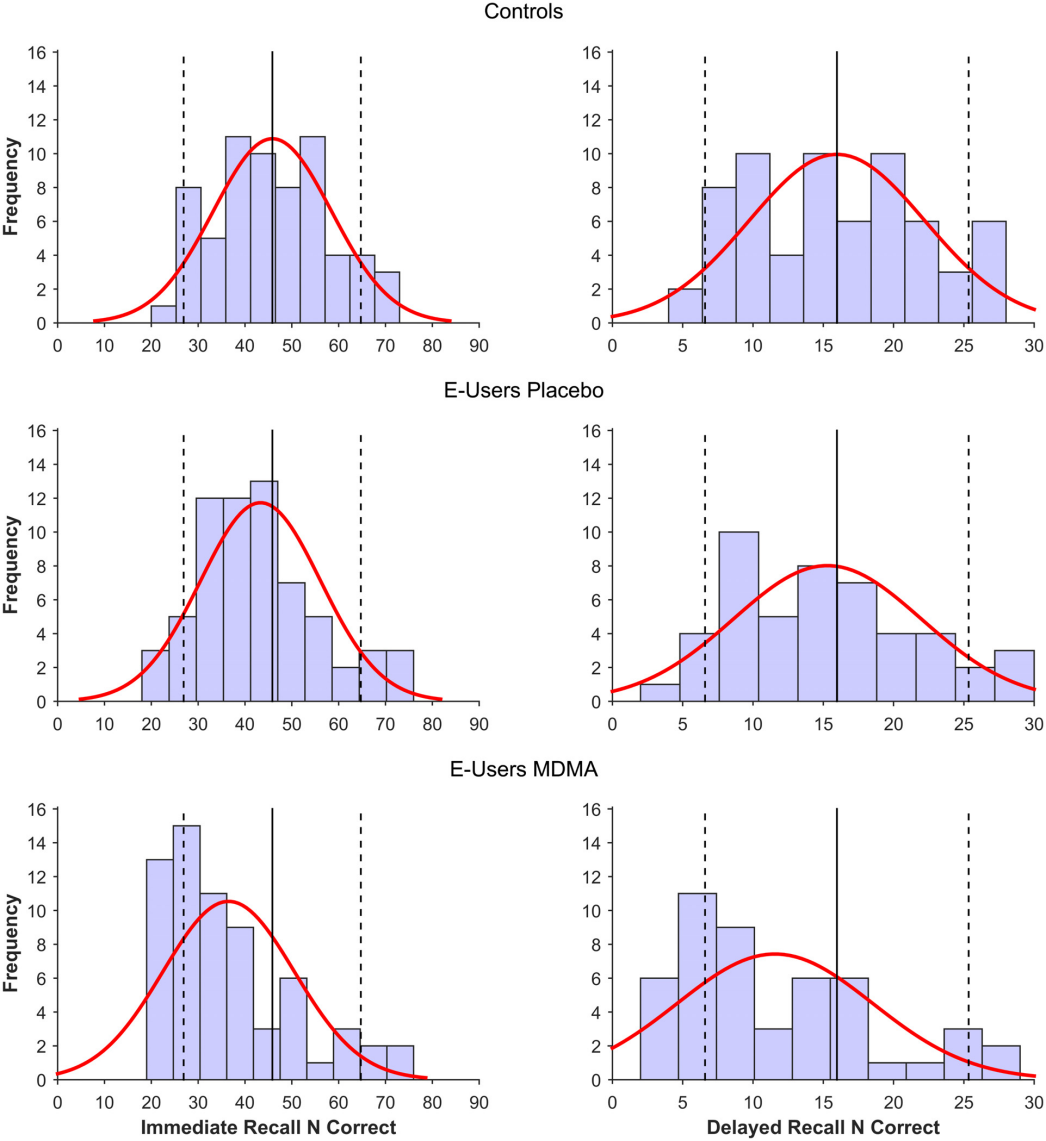
Frequency distribution of the raw IR (left panel) and DR (right panel) scores in the Ecstasy users during MDMA intoxication and placebo, and healthy controls. The straight line represents the mean IR (left) or DR (right) score in the healthy control group, the dotted lines represent the memory criterion, that is, 1.5 SDs of the healthy control group. Participants that fall on the left side of the left dotted line have impaired memory relative to the criterion; participants that fall on the right side of the right dotted line have superior memory performance relative to the criterion.

The logistic regression analyses showed that Ecstasy use *per se* did not statistically contribute significantly to the odds for having clinically deficient immediate recall (OR = 2.63, 95% CI [0.49, 14.05], *p* = .26) and delayed recall (OR = 0.67, 95% CI [0.06, 7.61], *p* = .75). Being an E-user, therefore, did not significantly predict having clinically relevant memory impairment (Fig 2).

#### Ecstasy exposure and memory impairment

Logistic regression analyses with clinical impairment as the dependent variable and times used as the regressor showed that ‘times used’ did not significantly contribute to the odds for having clinically deficient immediate recall (OR = 1.01, 95% CI [0.99, 1.02], *p* = .17) and delayed recall (OR = 0.72, 95% CI [0.34, 1.54], *p* = .40). The logistic regression analyses including ‘years of use’ showed that this predictor also did not contribute to the chance of having clinically significant IR (OR = 1.02, 95% CI [0.69, 1.50], *p* = .94) and DR memory impairment (OR = .66, 95% CI [0.15, 2.80], *p* = .57).

### Short-term or acute effects of MDMA

#### MDMA intoxication and memory

LMEM analyses revealed significant main effects of Treatment on IR Score [*F* (1, 65.5) = 18.90, *p* < .001, *η*^*2*^*G =* .*06*]) and DR Score [*F* (1, 48.7) = 21.55, *p* < .001, *η*^*2*^*G =* .07]. Participants recalled on average 6.90 fewer words during the immediate recall, and 3.87 fewer words during the delayed recall, in the MDMA condition, compared to the placebo condition (Fig 1). Logistic regression with clinical impairment as the dependent variable and treatment (MDMA, placebo) as the regressor revealed that the impairment was also of clinical relevance; MDMA intoxication contributed to the chance of having clinically deficient memory performance during immediate recall (OR = 0.20, 95% CI [0.07, 0.58], *p* = .003) and delayed recall (OR = 0.06, 95% CI [0.01, 0.51], *p* = .01) (Fig 2).

#### MDMA disposition and memory

LMEM analyses with MDMA blood concentration as the dependent variable and clinical impairment (based on IR scores) as a fixed effect showed no significant effect of clinical impairment [*F* (1, 64.81) = 1.17, *p* = .28, *η*^*2*^*G* = .04]. Bayes factor *t*-tests revealed only anecdotal evidence for the null hypothesis for differences in MDMA blood concentration between clinical relevance groups (*BF*_*01*_ = 2.03 ± 0.02%). Therefore, there is weak evidence that clinically relevant memory impairments for immediate recall while under the influence are not associated with a greater blood concentration of MDMA. These analyses could not be performed for DR memory impairments because MDMA concentrations for those with clinically relevant DR memory impairment were missing.

## Discussion

The central question of the present study was whether being a light E-user increases the chance of having clinically relevant verbal memory impairment. Results showed that memory performance of E-users did not differ from that of matched controls and that it was well within the normal range. Quantifiers of lifetime Ecstasy use (e.g., times used, years of use) did also not increase the chance of having clinically deficient memory performance.

The absence of a persistent long-term effect of Ecstasy on memory performance might be attributed to a difference in characteristics of the groups included in the present study and those of previous studies. Rogers (2009) interestingly pointed at the fact that in previous retrospective studies, samples were not drawn from comparable populations and that Ecstasy–using groups were on average less intelligent than their controls and tended to perform worse on neurocognitive tasks [24]. In the present pooled data-analysis, samples were drawn from comparable groups. Participants in the studies were recruited using the same strategies and in the same places (e.g., adverts at university and on a website digiprik.nl). All of the participants with known ‘current occupation’ details were students. Second, E-users in the present study had a low lifetime use history. Conceivably, the present sample was less exposed to potentially toxic substances, as being light E-users, and accordingly light polydrug users. The interaction effects of Ecstasy/MDMA with other co-consumed substances (e.g., alcohol, nicotine, cannabis) has been understudied, though it has been hypothesized that it could lead to increased toxicity and/or higher chance at compulsive use [47]. If this is indeed the case, more cognitive and behavioral problems would be expected in a group with heavy use and co-use of substances. In addition, light E-users probably haven’t been subjected to ‘aggregation toxicity’ to such an extent as heavy users have. This term ‘aggregation toxicity’ was coined by amphetamine researchers, almost 70 years ago, and refers to the fact that environmental factors linked to party-settings like high ambient temperature, crowding, extensive dancing and little fluid intake, play a large role in the severity of neurotoxicity. This was at least shown in animal models, that is, preventing acute hyperthermia attenuated the long-term MDMA toxicity [48].

An alternative explanation may relate to task complexity. Previously, Brown and colleagues have shown that memory tasks of higher complexity [49] showed reliable memory deficits in ‘moderate’ E-users. According to the criteria of the authors, our task would be classified as a ‘simple explicit memory’ task and therefore no differences between groups would be expected. However, Brown et al. (2010) did not test this assumption nor did they establish whether low-use E-users were impaired in tasks of higher complexity. Furthermore, they only reported statistical significance levels and did not address clinical significance. In contrast, Bedi and colleagues (2008) showed that evidence for impairing effects of Ecstasy on higher-level cognitive functions in their study was weak after controlling for potential confounds, and moreover not deemed to be of clinical significance [50].

A secondary question addressed in this study was whether MDMA intoxication leads to statistically and clinically significant memory impairment. Findings showed that it did. Where the statistical significance was a confirmation of the findings from the pooled studies [15–17, 19], the proof of clinical relevance was new. In addition, it was shown that MDMA blood concentrations during intoxication did not contribute to the chance of having clinically deficient memory performance. More specifically, the level of exposure to this ‘toxic’ substance is not a determinant of the chance of having clinically significant memory impairment; it is just the exposure per se. It should be noted however that the range of MDMA blood-concentrations was very small because all studies employed a single and identical dose, namely 75 mg. Our findings, therefore, do not exclude the possibility that memory impairment will increase with higher MDMA doses and/or blood-concentrations. Interestingly, the combination of the short-term and long-term findings in the present study demonstrates that while clinically deficient memory performance is present during intoxication, it is absent during abstinence. Together, this could indicate that light Ecstasy use does not lead to clinically deficient memory performance in simple explicit memory tasks in the long run. Preclinical research has also shown that the acute MDMA effects on memory and learning are more extensive than the chronic effects [51], although these studies have generally focused on the neurotoxic potential of MDMA after high, non-human equivalent doses.

A potential limitation of the present study relates to the type of included user, that is, no heavy users but only light (polydrug) E-users participated. Therefore results only pertain to this particular group. On the other hand, not all E-users are heavy users and consequently the general message about long-term effects must be nuanced and might not apply to light users. Another limitation is that information on premorbid intelligence levels was not systematically gathered in the pooled studies. Such information on baseline intelligence could provide additional evidence for how MDMA may affect memory. However, statistical tests did show that memory performance was similarly distributed for E-users when abstinent and the controls. Participants in the pooled studies could therefore be regarded as similar in terms of cognitive abilities. A third potential limitation is that we only collected data on the lifetime use and not on the amount (number of tablets) of ecstasy/MDMA used. Gallagher and colleagues argued that the number of tablets used is a better predictor than the estimated total lifetime use. They showed that the typical ecstasy dose consumed in a single session is an important predictor of prospective memory impairments with higher doses giving rise to greater impairment [52]. However, when knowing the amount of tablets taken, the exact dose of MDMA is still unknown. A fourth potential limitation refers to the fact that only the Word Learning Test was used. As mentioned above, it has been shown previously by Brown and colleagues that more complex measures show impairment in E-users compared to drug naïve controls [49]. However, in that case, the clinical significance of effects also should be taken into account. A fifth potential limitation is linked with the approach used by the present study, i.e. we do not consider baseline levels of performance and it cannot be excluded that subtler deficits have an impact on daily life activities in case this activities tap onto the assessed skill.

Future studies looking into long-term effects of Ecstasy use should gather additional genetic information as it has been shown that specific genes in combination with life-time use characteristics differentially influence memory performance in E-users [53]. Another related example is genes determining metabolism rate and, consequently, neurotoxicological potential of MDMA. Even though it was shown previously that one gene (CYP2D6) that is highly involved in metabolic clearance of MDMA was of lower clinical relevance than previously predicted [54], more research into similar genes could provide additional information on the relation between these and clinically relevant behavioral outcomes. Furthermore, preclinical studies have shown that MDMA is able to influence the expression of serotonin-related genes [55, 56] which might manifest as cognitive problems. So, next to genes, epigenetics will provide a more detailed view on the mechanisms underlying potential impairment observed in E-users. Besides genetics, it would be informative to include data on environmental circumstances linked to drug use history (e.g., ‘aggregation toxicity’ [48], or taking ‘protective’ substances before/after use [57]) to investigate the contribution of these factors to cognitive performance. However, like drug history information, this information would be retrospective and subject to recall-bias; nevertheless, it could be used as a covariate in statistical analyses (e.g., LMEM) next to other demographic variables. Furthermore, inclusion of tasks of different complexity and brain imaging measures could provide a more detailed picture on the potential impairing effect of chronic Ecstasy use. It has been shown that behavioral measures can be less sensitive than imaging measures in that aberrant functional patterns are shown in the absence of behavioral effects (e.g., [20, 21]). However, with regard to the behavioral measure (verbal memory) used in the present study it is interesting to note that at least in healthy older adults, performance in immediate verbal memory was the best predictor for activities of daily living [38]. This suggests that the outcomes in the Word Learning Task could also be predictive of functional outcomes in daily life in the present sample of drug users. In conclusion, while the question of clinical relevance of impairment is rarely addressed in studies evaluating the long-term effects of drug use. The present study did address clinical relevance and showed that light use of Ecstasy/MDMA does not lead to clinically deficient memory performance on the long-term, that is, after 1–12 years of use. Future studies should adopt the combined approach of determining clinical relevance together with statistical significance. Additionally, it has to be investigated whether the current findings apply to more complex cognitive measures in diverse ‘user categories’ using a combination of genetics, imaging techniques and neuropsychological assessments.
